# MPSS profiling of human embryonic stem cells

**DOI:** 10.1186/1471-213X-4-10

**Published:** 2004-08-10

**Authors:** Ralph Brandenberger, Irina Khrebtukova, R Scott Thies, Takumi Miura, Cai Jingli, Raj Puri, Tom Vasicek, Jane Lebkowski, Mahendra Rao

**Affiliations:** 1 National Institute on Aging; GRC; Laboratory of Neuroscience, 5600 Nathan Shock Drive; Room 4E02; Baltimore, MD 21224, USA; 2 Geron Corporation, 230 Constitution Drive, Menlo Park, CA 94025, USA; 3 Lynx Therapeutics, Inc. 25861 Industrial Blvd., Hayward, CA 94545, USA; 4 Laboratory of Molecular Tumor Biology, Division of Cellular and Gene Therapies, Center for Biologics Evaluation and Research, Food and Drug Administration, Bethesda, MD 20892; 5 Department of Neuroscience, School of Medicine, Johns Hopkins University, Baltimore, MD 21205

## Abstract

**Background:**

Pooled human embryonic stem cells (hESC) cell lines were profiled to obtain a comprehensive list of genes common to undifferentiated human embryonic stem cells.

**Results:**

Pooled hESC lines were profiled to obtain a comprehensive list of genes common to human ES cells. Massively parallel signature sequencing (MPSS) of approximately three million signature tags (signatures) identified close to eleven thousand unique transcripts, of which approximately 25% were uncharacterised or novel genes. Expression of previously identified ES cell markers was confirmed and multiple genes not known to be expressed by ES cells were identified by comparing with public SAGE databases, EST libraries and parallel analysis by microarray and RT-PCR. Chromosomal mapping of expressed genes failed to identify major hotspots and confirmed expression of genes that map to the X and Y chromosome. Comparison with published data sets confirmed the validity of the analysis and the depth and power of MPSS.

**Conclusions:**

Overall, our analysis provides a molecular signature of genes expressed by undifferentiated ES cells that can be used to monitor the state of ES cells isolated by different laboratories using independent methods and maintained under differing culture conditions

## Background

Multiple large-scale analytical techniques to assess gene expression in defined cell populations have been developed. These include microarray analysis, EST enumeration, SAGE and MPSS. Each of these techniques offers unique advantages and disadvantages. Technique selection largely depends on the expertise of the investigator, the cost, the availability of the techniques, the amount of RNA/DNA that is available, and the existence of the genome databases. The human genome dataset is the best annotated one available [1,2]- making large scale gene expression analysis of human tissues and cells uniquely fruitful for investigators due to the increased ability to identify full length transcripts with predicted gene function instead of EST's.

Human ES cells have been isolated relatively recently and ES cell genes are underrepresented in current databases. More importantly, recent evidence has suggested that mouse ES and human ES cells differ significantly in their fundamental biology [3,4] and one cannot readily extrapolate from one species to another. However, comparing results between species may provide unique insights. Given the wealth of SAGE and microarray data available from rodent ES cells examining human ES cells with similar techniques as has been done recently by several investigators [3-11] should be very useful in furthering our understanding of this special stem cell population. Until recently however, it has been difficult to obtain RNA from a homogenous population of undifferentiated hESC for such an analysis as cells could not be grown without feeders and few unambiguous ES cell markers had been described. However, we and others have now described markers that will clearly assess the state of ES cells using a combination of immunocytochemistry and RT-PCR [3,12,13] In addition, techniques of harvesting ES cells away from feeder layers have been developed and verified (our unpublished results) and methods of growing ES cells without feeders have been described [14]. These techniques, have allowed us (and others) to obtain large amounts of validated RNA/cDNA samples for comparison by microarray [3-11], SAGE [8] or EST enumeration [9].

We selected MPSS for this analysis as it offers some unique advantages over other methods including SAGE [15,16]. MPSS offers sufficient depth of coverage when over one million transcripts are sequenced [16] and is efficient, as the numbers of sequences obtained are an order of magnitude larger than with shotgun sequencing or SAGE. It is relatively rapid with a turnaround of a six to ten weeks, and if done with human tissues, more than 80% of transcripts can be mapped to the human genome with current tools. Further, independent analysis has suggested that expression at greater than 3 tpm (transcripts per million) is predictive of detectable, reliable expression, equivalent to roughly one transcript per cell – a sensitivity that is unparalleled when compared to other large-scale analysis techniques [16]. Finally, MPSS libraries can be translated into SAGE libraries and compared to existing SAGE library sets using freely available tools such as digital differential display, allowing ready comparisons to existing SAGE/MPSS libraries of mouse ES cells. It is important to note that we found 14 base pair SAGE tags are generally not as specific as 17 base MPSS signatures and that SAGE sampling depth is usually insufficient. Newer technologies such as extended sequencing to 20 base pairs in MPSS, 24 base pairs in SAGE or cheaper bead alternatives such as those described by Illumina may offer additional depth of coverage and a cheaper price but these at present remain limited in availability.

We have utilized MPSS using a pooled sample of three human ES cell lines grown in feeder-free culture conditions over multiple passages [17,18] to assess the overall state of undifferentiated ES cells. Our rationale for using pooled sample rather than individual samples was based on the fact that no standardized medium and culture conditions have been established for growing and propagating ES cell lines. Variation observed by sampling single lines may be due to culture conditions rather than intrinsic differences. We reasoned therefore that a need existed to establish a reference baseline using pooled samples to enhance the similarities and provide evidence for candidate genes that should be examined for differences such as expression of HLA genes, Y chromosome and X chromosome genes, imprinted genes and genes regulating the methylation state. Our results show that MPSS provides a greater depth of coverage than EST scan or microarray and provides a comprehensive expression profile for this stem cell type. The data set generated allows us and others to identify multiple genes that were not previously known to be expressed in this population, including novel gene as well as obtain a global overview of pathways that are active during the process of self-renewal.

## Results

### MPSSS analysis of pooled samples

**Figure 1:**
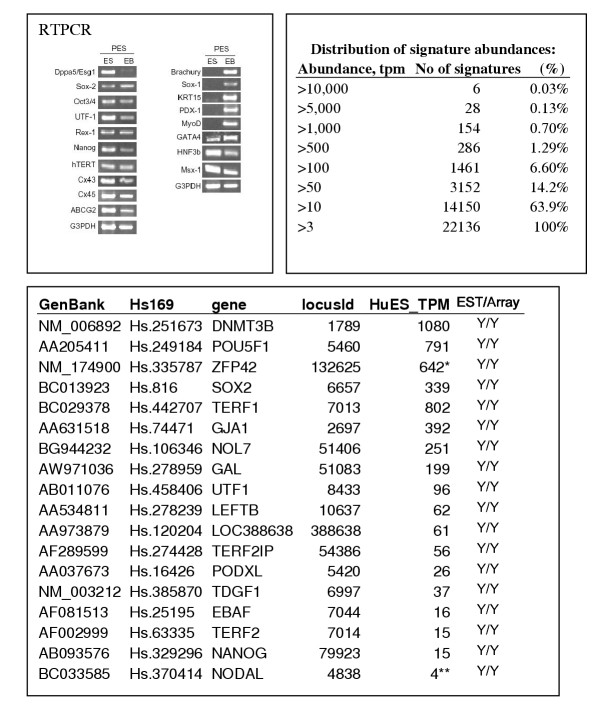
RT-PCR analysis (a), cumulative tpm (b) and tpm of known ES cell markers (c) is shown. Note that MPSS identifies most known markers of huES cells and expression is at high tpm levels. * – signature maps to >100 location in the genome (class 0); ** – artifactual (class 5) signature

A pooled sample of undifferentiated human ES cell lines H1, H7, and H9 grown in feeder-cell free conditions [19] was used for the preparation of mRNA as previously described [20]. Growth without feeders avoids complication from feeder contamination, which even with good harvesting techniques [14,21] ranges between 1–3% (unpublished data) and is sufficient to be detected by MPSS (Dr. B. Lim-Harvard University personal communication). Under these conditions, 80–95% of the cells express SSEA-4, 91–94% express TRA-1-60, and 88–93% express TRA-1-81, previously described markers for undifferentiated hESC [19]. Microarray analysis of 2802 genes suggests that these cells are remarkably similar in their gene expression profiles, with only 5 genes being more than 2-fold different between the three cell lines [17,18] (and data not shown). The undifferentiated state of the cells was also assessed by RT-PCR of known markers of undifferentiated hESC on mRNA of the pooled hESC sample (Figure 1). In addition absence of early markers of differentiation was assessed. No expression of GATA, Sox-1, nestin, Pdx-1 or markers of trophoectoerm were detected in samples used (Supplementary table 3a, see also 3)

Pooled mRNA of the three hESC lines was subjected to MPSS analysis at Lynx Therapeutics (Hayward, CA), generating 22,136 distinct and significant signature sequences from a total of 2,786,765 sequences (see Methods and additional file 1). Each signature was ranked, as outlined in Methods (Table 1), based on its position and orientation within the transcript, and the presence of a polyadenylation signal and polyA in the transcript sequence. 16,675 signatures (75%) mapped to UniGene transcripts; 40 signatures (0.2%) mapped to mitochondrial transcripts; 3,818 signatures (17%) matched genomic sequences but did not map to a UniGene cluster; 927 (4%) signatures matched sequences present at more than 100 genome locations (class 0, representing transcripts containing repetitive elements in their 3' UTR). 676 (3%) signatures did not match to genome or UniGene sequences. Some UniGene clusters contain multiple signatures. These signatures likely represent either transcripts of alternative termination sites, or artefacts of MPSS library construction. Signature classification helps to distinguish artifactual signatures from signatures representing expressed transcripts. For example, signatures of class 1 to 3 are 3'most signatures in mRNA or EST sequences with poly (A) signal and/or polyA tail and most likely represent transcripts with multiple polyadenylation sites. Artifactual signatures constituted 1–3% of the tpm count of the "real" signature, although occasionally close counts were observed (data not shown; see supplementary data tables, additional files 2, 3). To simplify the MPSS data analysis and pair-wise comparison of ES cell data from this study to other datasets, multiple signatures mapping to the same Unigene ID (Hs build 169) were combined into one tpm count as the sum of tpm for signatures of class 1, 2, 3, 22, 23 if any found. These are 3'most signatures close to polyA signal and/or polyA tail, most probably representing true transcripts with alternative termination. If no signatures of above classes were found, then sum of class 4 (3'most, no polyA features) was used. If none the above, the sum of class 5 signatures was used for the tpm calculation per unigene cluster. Resulting table containing data for 8679 unigene clusters, 11 mitochondrial genes, and including 1991 signatures that did not map to unigene but uniquely matched genomic sequences (potential novel transcripts), is presented in supplementary table (additional file 4) and available for download from Lynx [27].

**Table 1:** Classification of the MPSS cDNA signatures. The signature classification used for annotation is shown * The Class 0 signatures are the signatures that hit genome more than 100 times, which is treated as a "repeat sequence". ** The polyA tail is defined as a stretch of A's (at least 13 out of 15 bases) that is no more than 50 bases away from the end of the source sequence. The polyA signal is either AATAAA or ATTAAA that has at least one base within the last 50 base before the end of the source sequence or the polyA tail. *** All the virtual signatures extracted from the genomic sequences are classified as class 1000 signatures.

**Virtual Signature Class**	**MRNA Orientation**	Poly-Adenelation Features **	**Position**
0*	Either – Repeat Warning	Not applicable	Not applicable
1	Forward Strand	Poly-A Signal, Poly-A Tail	3' most
2		Poly-A Signal	3' most
3		Poly-A Tail	3' most
4		None	3' most
5		None	Not 3' most
6		Internal Poly-A	Not 3' most
11	Reverse Strand	Poly-A Signal, Poly-A Tail	5' most
12		Poly-A Signal	5' most
13		Poly-A Tail	5' most
14		None	5' most
15		None	Not 5' most
16		Internal Poly-A	Not 5' most
22	Unknown	Poly-A Signal	Last before signal
23		Poly-A Tail	Last before tail
24		None	Last in sequence
25		None	Not last
26		Internal Poly-A	Not 3' most
1000***	Unknown – Derived from Genomic Sequence	Not applicable	Not applicable

**Figure 2:**
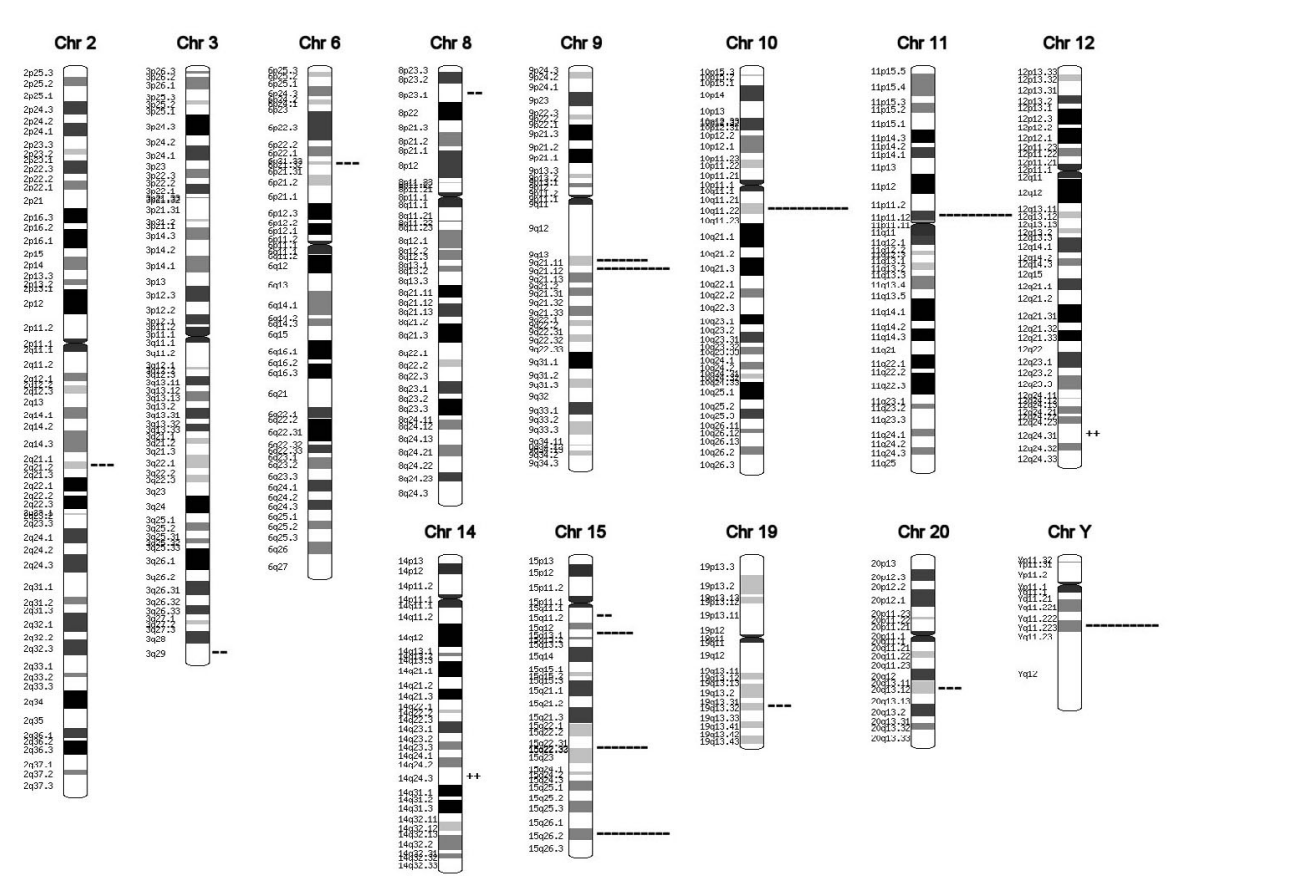
Cytoband mapping of ES cell expressed genes and regions of relatively high and low transcription relative to the refseq database is shown. More detailed mapping information is presented in supplementary tables.

The frequency distribution of the signatures shows that the 200 most abundant signatures represent 99% of the total number of signature counts obtained from the hESC (Figure 1). Most of top 200 genes (unigene clusters, additional file 5) represent ribosomal genes and genes involved in protein and nucleic acid synthesis and are consistent with results obtained by EST scan and other analyses (data not shown, and [5,8,9]). We note that several ribosomal genes were identified as being overexpressed by microarray, SAGE and EST scan as well (see additional files 16, 17, 18). Comparison of the pattern of gene expression with other cell types showed a very similar expression profile with housekeeping genes being the predominant population of sequences in all cell types examined (data not shown). Only three known ES cell specific genes were present in the top 200 genes (additional file 5 and Figure 1). These included SOX-2, DNMT3β, and Oct-4. As in other cells cell type specific genes, transcription factors and cytokines were present at much lower abundance (<50 tpm on average). These low tpm level genes were often not detected by other methods (discussed below). The expression level of cell surface receptors for fibronectin are high (ITGB1 – 578 tpm) and their presence was confirmed by immunocytochemistry and RT-PCR, suggesting that feeder-free clones may grow well on this substrate (data not shown, see also Figure 2 and [14,21]). The major signaling pathways represented in the top 200 most abundant genes are the FGF signaling pathway, with FGFR1 being most abundant (673 tpm, Figure 2), and the ras activated pathway, with two members of the ras family (NRAS-related and ran) being present in the top 200. This is consistent with data that E-Ras is critical for rodent ES cell self-renewal [22]. No transcripts for HRASP (Homologue of ERAS pseudogene) were detected however (Figure 2), suggesting that these other ras family members may subserve this critical role of self-renewal [9]. The absence of E-Ras was confirmed by RT-PCR (data not shown), as was the presence of FGFR1 (Figure 2, [22], and data not shown).

**Figure 3:**
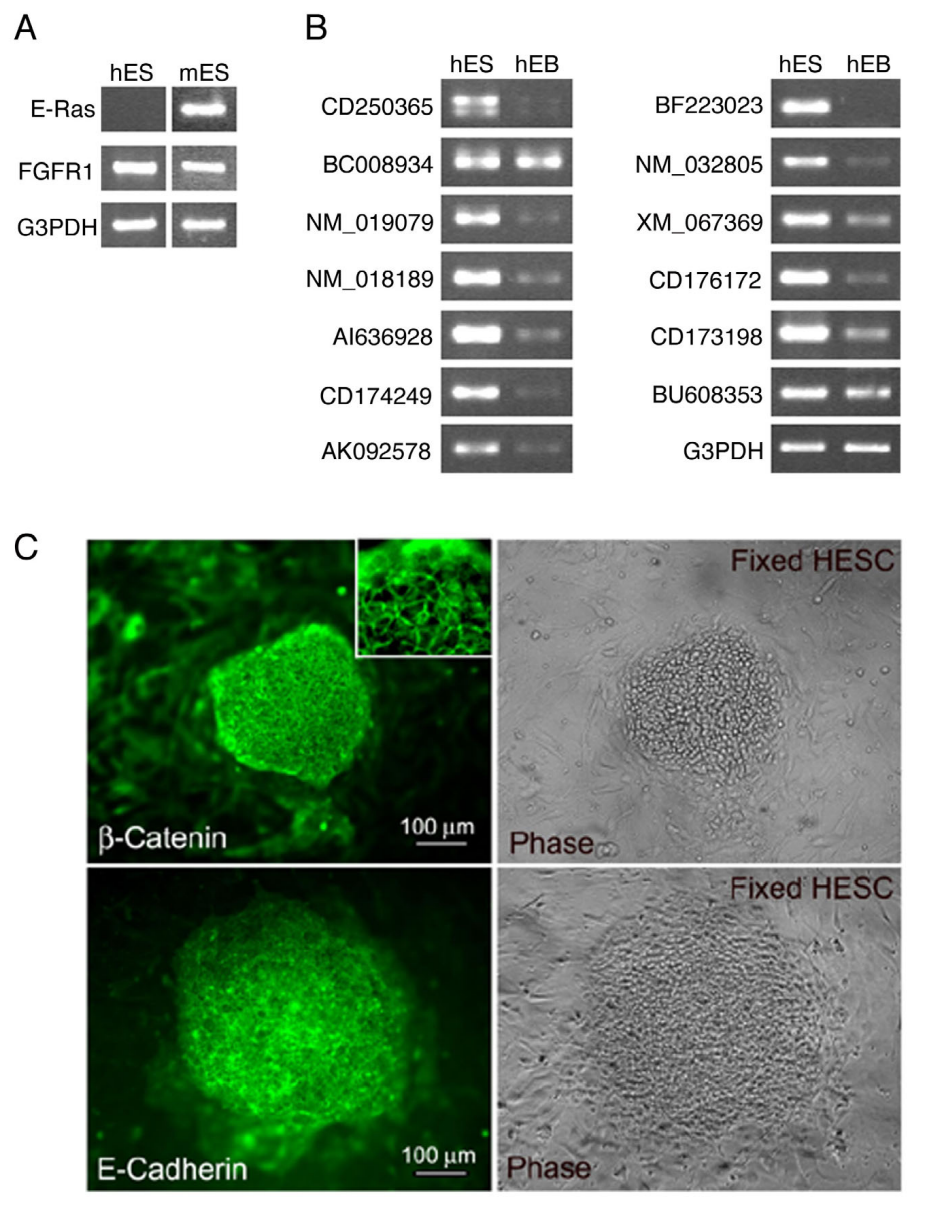
RT-PCR for E-ras/RASP, FGFR1 and novel genes identified as enriched in undifferentiated ES cells is shown in Panel A and B. Localization of E-cadherin and β-catenin in undifferentiated ES cell is shown in Panel C. All of the genes identified by MPSS and tested were present in undifferentiated ES cells and most were significantly downregulated as cells differentiated. Note the high expression at the cell surface and low or undetectable levels of β-catenin in the nucleus.

### Major pathways present at detectable levels by MPSS

To gain a broad overview of the properties of hESC, we mapped the genes found in the hESC cells to the human genome to get an overview of the chromosomal distribution of genes expressed in hESC (Figure 3 and additional files 6, 7, 8, 9, 10, 11). Overall, MPSS detected gene expression in most of the previously identified zones of transcriptional activity within chromosomes. Two chromosomal regions contained more genes expressed in hESC – than expected, and several regions where fewer genes were expressed, compared to the total number of genes located within a particular chromosomal region. No bias to chromosome 17, 12 or X was seen either in overall gene expression or in a particular cytoband. The failure to detect a bias was confirmed by mapping EST scan data [8] as well. The overall distribution patterns were similar and did not show any bias at this level of resolution. Interestingly, gene expression from both X and Y chromosomes was observed. Unlike rodent ES lines both male and female ES lines have been obtained with roughly equal frequency [20] suggesting that when individual cell lines are examined differences between levels of expression between male and female will be present and detectable.

Likewise, MPSS detected expression of several MHC Class I and II genes, suggesting that MPSS can identify differences between ES cell samples when HLA gene expression is used to type cells [17,18]. We also note that both H19 and Igf2 were expressed at detectable levels. H19 and Igf2 are located adjacent to each other on chromosome 11p15.5 and are reciprocally regulated by imprinting, H19 being paternally imprinted, and IGF2 being maternally imprinted [23,24]. It is therefore likely that their ratio of expression is likely to differ between cell populations and may represent a simple assessment of the imprinting status of cells.

We classified genes expressed into ECM related, homeobox containing, zinc finger proteins, novel genes as well as genes which could assigned to major signaling pathways such as wnt, BMP/TGFβ, LIF, receptors, etc. This data is provided in excel files in the supplementary information provided (additional files 12, 13). Overall certain general themes emerged when genes were classified into such a fashion. We find that: A) hESC express markers characteristic of ES cells in general and few markers characteristic of differentiated cells confirming the initial purity of ES cells used for this analysis and the fidelity of the analysis B) Ribosomal protein transcripts, and mitochondrial genes are highly expressed in ES cells (relative to other transcripts) and constitute more than 50% of the total transcripts analyzed (Figure 1, additional files 5, 16, 17, 18). And this is similar to other samples analyzed [3-11], (Lynx Inc. data not shown) C) Positive regulators of the cell cycle, TERT and antisenescence related genes and DNA repair pathway regulators are expressed at high levels while proapototic genes, Rb and p53 pathways regulators are expressed at low levels (see table 2 for an example of TERT related gene expression, see supplementary tables (additional files 12, 13) for cell cycle, apoptosis and other pathways) D) The number of novel genes or genes of unknown function is high (2600/11,000) and constitutes approximately 25% of the unique signatures (see additional file 13 for a listing of genes of unknown function, their chromosomal mapping, and UniGene identity). Comparison with other samples suggest that the number of novel genes or genes of unknown function seen are higher in ES cells (25% versus 20%). E) Components of most major signaling pathways are present but so are negative regulators (including zinc finger proteins), suggesting that inhibition plays an important role in maintaining cells in an undifferentiated state (see additional file 13).

**Table 2:** Senesence and Aging related genes A subset of genes related to senescence and aging that may regulate the lack of senescense in ES cells is shown. Note that the telomerase, morf's, nortalins and sirtuins are all expressed in ES cells. *The TERT gene has a signature uniquely mapping to an intron (cryptic exon?), which was present in all runs of the ES cell analysis and was not found in other human samples (not shown).

**HuES_TPM**	**Gene Bank**	**Hs169**	**Gene**	**chr**
802	BC029378	Hs.442707	TERF1	8q13
56	AF289599	Hs.274428	TERF2IP	16q23.1
38	AI742882	Hs.409194	TNKS	8p23.1
15	AF002999	Hs.63335	TERF2	16q22.1
10	AW271065	Hs.9645	TNKS1BP1	11q12.1
9	BC005030	Hs.7797	TINF2	14q11.2
7	AF264912	Hs.280776	TNKS2	10q23.3
10*	NM_003219	Hs.439911	TERT	5p15.33
321	NM_004134	Hs.184233	HSPA9B	5q31.1
94	AF070664	Hs.374503	MORF4L1	15q24
80	BC017305	Hs.528641	SIRT7	17q25
42	AF100620	Hs.411358	MORF4L2	Xq22
27	NM_012238	Hs.31176	SIRT1	10q22.1
10	BM803485	Hs.511950	SIRT3	11p15.5
16	AL579291	Hs.282331	SIRT5	6p23

Examination of signaling pathways suggest that wnt, TGFβ and FGF signaling pathways are likely important in regulating the ES cell state while LIF/gp130 signaling is not as important. These conclusions are based on examining the expression of the positive and negative regulators of a particular pathway by MPSS, and EST scan. When critical components are low or absent we have tentatively assumed that the pathway is unlikely to be active. An example of the Igf/PTEN pathway is shown to illustrate the logic (Table 3) and other pathways along with verification with EST scan are summarized in the supplementary tables (additional files 12, 13). Note the high levels of soluble frizzled receptors and the expression of E-cadherin (negatively regulating β-catenin translocation). The expression of cadherin and β-catenin was confirmed by immunocytochemistry (Figure 2). The relatively fidelity of the conclusion was confirmed by examining the expression of E-cadherin by immunocytochemistry and localizing β-catenin expression.

**Table 3:** IGF-/PTEN/Akt and Ras/Raf/MAP pathway A subset of genes related to Igf/PTEN pathway that are expressed in undifferentiated ES cells is shown. Note that the overall pattern of expression suggest that this pathway is active in undifferentiated ES cells.

**Tpm ES**	**Tpm EB**	**Unigene ID**	**Locus ID**	**Description**
14	32	Hs.239176	3480	IGF-1 receptor
N.D.	N.D.	Hs.390242	3667	IRS-1
0	0	Hs.253309	5728	PTEN
N.D.	N.D.	Hs.32942	5294	PI3K
11	8	Hs.433611	5163	PDK1
0	15	Hs.92261	5164	PDK2
N.D.	N.D.	Hs.6196	3611	ILK
75	157	Hs.368861	207	AKT1
15	82	Hs.170133	2308	FKHR (FoxO1A)
78	54	Hs.14845	2309	FKHRL1 (FoxO3A)
15	88	Hs.282359	2932	GSK3beta
39	14	Hs.238990	1027	p27
280	240	Hs.371468	595	Cyclin D1
0	594	Hs.370771	1026	p21
1	10	Hs.329502	842	Caspase 9
0	39	Hs.76366	572	Bad
98	193	Hs.260523	4893	N-Ras
2	0	Hs.37003	3265	H-Ras
35	128	Hs.257266	5894	Raf1
N.D.	N.D.	Hs.132311	5604	MEK1
128	218	Hs.366546	5606	MEK2
37	75	Hs.324473	5594	ERK (p42 MAPK)

We compared the signature sequences detected in the hESC to an MPSS database of 36 human tissues and cell lines to look for genes that are unique to, or highly overexpressed in hESC. A list of several hundred was generated when a cutoff of 30 tpm or higher (ten fold above detection level) that were elevated in ES cells when compared to neural stem cells examined in a similar manner was used. This list is provided in supplementary materials (additional file 14). A list of 13 highly enriched genes of unknown function is shown in Table 4, and the tpm values for the corresponding signatures in each of 36 tissues or cell lines is provided in the supporting information (additional file 15). The expression in ES cells, of these 13 genes was confirmed by designing PCR primers to different regions and examining gene expression (Figure 2). Several of these genes are highly expressed in hESC and absent in most other tissues tested (Table 4, additional file 15, and data not shown), are downregulated as ES cells differentiate (Figure 2), and are good novel, candidate markers for undifferentiated hESC.

**Table 4:** Novel genes enriched in hESC as assessed by MPSS A short list of genes of unknown function that are highly enriched in three ES cell lines comparing to 36 different tissues and cells are shown. A complete list of unknown genes expressed in pooled hESC cells is presented in supplementary tables. * NS-neural stem cells, TH-thymus, HY-hypothalamus, PG-pituitary gland, TE-testis ** this gene (Hs.507833 in the unigene Hs.169) is transcribed in antisense to HDCMA18P (Hs.278635)

**SIGNATURE**	**HuES,TPM**	**Chr**	**GB:description**	Other 36, TPM*
GATCTCCAGTAGACTTA	1646	4	CD250365:Homo sapiens transcribed sequence **	**NS-10**
GATCTGTTAACAAAGGA	967	16	BC008934:claudin 6	**ND**
GATCTAGAAGTTGCAAC	489	1	NM_019079:hypothetical protein FLJ10884	**ND**
GATCTTTTTTTTTGCCC	455	3	NM_018189:hypothetical protein FLJ10713	**TH-47, HY-3, PG-3**
GATCCCCATCCAAAAGA	366	7	AI636928:Homo sapiens transcribed sequences	**MCF7-2**
GATCCACCTAGGACCTC	244	X	CD174249:Homo sapiens transcribed sequence	**ND**
GATCCGCCTCCTTGGCC	240	4	AK092578:Sapiens cDNA FLJ35259 fis	**ND**
GATCCTAGCCAAGCCCC	169	3	BF223023:Homo sapiens transcribed sequences	**ND**
GATCTGGCCCGCCACCA	150	16	NM_032805:hypothetical protein FLJ14549 (ZNF206)	**ND**
GATCGTTGTGGTGGACT	146	3	XM_067369:similar to Heterochronic gene LIN-41	**ND**
GATCCACCACATGGCGA	92	11	CD176172:Homo sapiens transcribed sequence	**ND**
GATCCAACAATTCTACT	78	U	CD173198:Homo sapiens transcribed sequences	**TE-33**
GATCTTCTAAACCCATC	75	12	BU608353:Homo sapiens transcribed sequence	**ND**

### Comparing with other data sets

Recently we and others have begun examining hESC with EST scan [10] and microarray analysis to develop a characteristic profile of this unique population [3-10]. We used this data to compare the sensitivity of MPSS with EST scan and microarray analysis. We have previously reported a set of 90 genes reported common to 6 different hESC lines [10]. Of these, eighty-five were detected by MPSS showing a high degree of concordance (>90%). Of the five genes missing from the MPSS hESC data set, four of the genes had valid MPSS signatures (Table 5) and were readily detected in other human samples (data not shown). One gene (SNRPF) lacked a DpnII (GATC) site making it non-detectable by MPSS. GDF3 was detected at non-significant level in the hESC, though was detected by MPSS at higher level (10–30 tpm) in other ES cells tested (Dr. B. Lim-Harvard University personal communication, and additional file 17). Sperger et al., also used microarray to examine gene expression in undifferentiated cell lines [11]. They compared expression in undifferentiated cells with expression in EC carcinoma lines and with microarray data from several other cell lines. They have identified 895 genes (GenBank accession numbers) which reduce to 718 number of unigene identities when mapped to the unigene build Hs161. We have compared this data with the MPSS data and see that MPSS identified the large majority of these genes as well (additional file 16). Similar results were obtained when data was compared with that reported by Sato et al., and Abetya et al., [6,7] and a similar concordance in gene expression was observed (data not shown). Thus, MPSS provides an independent verification of the microarray results and in addition identifies other genes that may not be present on the arrays or detectable by current microarray techniques.

**Table 5:** MPSS tpm of genes reported as enriched by microarray in hESC Table 5 Tpm of genes identified as overexpressed microarray analysis of six pooled human ES cell lines. Note that most of them have high tpm values and are detected by MPSS. * – PSIP2 and PSIP1 have 3' alternate termination and distinguished by MPSS (but not by microarray); ** – PODXL: TPM for signature of class 5; 3' most signature has double palindrome and underrepresented. *** – higher expression of GDF3 was detected in other ES cells (suppl.table for BG02 and not shown). **** – expression detected in other human samples (not shown).

**GB_accession**	**Gene Symbol**	**HuES_TPM**
X85372	SNRPF	No GATC
NM_002295	LAMR1	6135
D23660	RPL4	5269
NM_001002	RPLP0	4656
NM_002520	NPM1	3207
X69391	RPL6	3745
M31520	RPS24	3183
AF070600	OK/SW-cl.56	2702
X57958	RPL7	1923
NM_024674	LIN-28/	1692
NM_145899	HMGIY	1618
NM_018407	LAPTM4B	1326
M94314	RPL24	1279
X62534	HMGB2	989
D13748	EIF4A1	1070
NM_006086	TUBB4	809
J04164	IFITM1	788
X69804	SSB	874
M93651	SET	1323
D00760	PSMA2	673
AL162079	SLC16A1	991
AF225425	SEMA6A	742
U28386	KPNA2	542
X74929	KRT8	543
NM_002300	LDHB	527
M97856	NASP	536
AF311912	SFRP2	457
AF020038	IDH1	450
D83174	SERPINH1	477
S74445	CRABP1	437
NM_000165	GJA1	392
AB040903	TD-60	524
AF063020	PSIP2*	389
U76713	HNRPAB	166
NM_000224	KRT18	302
NM_021144	PSIP1*	389
M94856	FABP5	257
NM_016304	Ribo 60S L30	247
AK094423	HNPRA1 like	214
AF055270	HSSG1 (SFRS7)	201
M77140	GAL	199
AF257659	CALU	100
AF098158	C20orf1	338
U41387	DDX21	179
AD001528	SMS	175
NM_006548	IMP-2	177
AJ223953	PTTG1	154
X54326	EPRS	210
D13627	CCT8	167
NM_012247	SEPHS1	306
D00762	PSMA3	123
AF005418	CYP26A1	121
M25753	CCNB1	168
NM_000884	IMPDH2	174
X16396	MTHFD2	113
NM_005159	ACTC	98
U31814	HDAC2	112
J04031	MTHFD1	104
NM_006341	MAD2L2	95
J03746	MGST1	88
NM_020997	LEFTB	62
M74091	CCNC	86
AK001962	BRIX	66
M36981	NME2	93
AL133611	Novel	63
X05360	CDC2	62
AB040930	LRRN1	46
AF071592	KIF4A	71
AF015254	STK12	41
X14253	TDGF1	37
AB023420	HSPA4	42
M19309	TNNT1	54
BC004200	PPAT	34
NM_024090	ELOVL6	23
NM_014366	NS	30
U97519	PODXL	26**
AF048722	PITX2	25
NM_024498	ZNF117	32
NM_001878	CRABP2	24
X59244	ZNF43	13
BC001068	C20orf129	17
NM_024865	Nanog	15
NM_024900	Jade-1	11
AB046793	KIAA1573	11
Z26317	DSG2	18
NM_020634	GDF3	1***
AF070651	ZNF257	0****
NM_016448	RAMP	0****
U88573	NBR2	0****
AB044157	GSH1	0****

Comparison with an EST scan analysis of 37,081 EST sequenced from a similar pooled sample of hESC [9,10] also showed a high degree of concordance. The EST scan analysis detected 8,801 distinct UniGene clusters in hESC versus 9,996 distinct UniGene clusters expressed at 4 tpm or higher in the MPSS dataset. Of the 8,801 UniGene clusters identified by the EST scan, 1,139 are singletons, i.e. identified by only one EST out of the 37,081 total EST's. 5,286 UniGene clusters have 5 or more ESTs as evidence, and only 118 UniGene clusters have more than 100 EST's as evidence. In contrast, all 9,996 UniGene clusters identified by MPSS were detected at 4 or more tpm and identified in multiple sequencing runs. More than 8,000 have at least 10 tpm, and over 1,000 have more than 100 tpm. Thus, although the EST's are longer in length and thus easier to assign to a particular gene, MPSS appears more sensitive than EST scan. MPSS for example identified almost twice as many genes as EST scan consistent with the difference in the depth of analysis (No of sequences MPSS/EST).

Richards et al [8] have used SAGE analysis to two ES cell lines. Their analysis revealed expression of approximately four thousand genes which was significantly fewer than that identified by MPSS consistent with the fewer number of gene tags sequenced. Comparison of the data sets however showed good concordance particularly for genes expressed at higher tpm levels. The entire comparison is presented in supplementary table (additional file 18) and is available for download from Lynx [27]. Overall MPSS could identify genes that other methods identified with an average concordance rate of 70%. The depth of analysis with MPSS at 2.4 million signatures however was significantly greater. MPSS in general identified many more genes than microarray or EST scan or SAGE (see above). The most direct comparison is with EST scan or SAGE, which do not rely on comparative gene expression to establish significance of gene expression. Overall our comparison suggests that MPSS results provide a complementary global overview of the transcriptome of the ES cell. The data supplement and extend the microarray, SAGE and EST scan data sets and provide an independent verification of the same. MPSS in addition identifies additional genes expressed particularly at lower tpm, that are either not present on microarrays or not detected with a lower resolution analysis.

## Discussion

Our results provide a global overview of the gene expression pattern of undifferentiated human ES cells and allow comparisons with other data sets. These results suggest the hESC are an actively dividing population of cells that exhibit high metabolic activity. Our analysis detected expression of approximately 10,600 unique transcripts, a figure that about a third of the total number of mapped genes. Unlike other cell types, however, a much larger fraction of unknown or novel genes was present. This high ratio likely represents the paucity of information available in existing libraries on this relatively newly characterized cell population rather than the possibility that ES cells use radically different pathways for self-renewal, survival, proliferation and differentiation.

Our results confirm the reported differences between rodent and human ES cells. We confirm the absence of expression of ERAS, Ehox and the orthologs PEPP1 and 2. The apparent lack of LIF requirement of hESC is reflected by the absence or low tpm levels for genes of the LIF pathway and high tpm for suppressors of LIF mediated signalling (see supporting information). The high level of expression of genes in the FGF pathway likely reflects the requirement of hESC for bFGF. The high level of FGFR1 expression suggests that FGFR1 is an important signal transducer and that FGF's other than FGF4 are important in hESC self-renewal. The high tpm of the fibronectin receptor also suggest that fibronectin or vitronectin are likely useful substitutes for matrigel and that activation of ras mediated signalling is likely critical, as has been described in the rodent ES cell analysis [20].

Comparing data from the MPSS analysis with microarray, SAGE and EST scan analyses suggest that MPSS is a powerful alternative to these techniques. MPSS identified virtually all of the genes highlighted as genes common between six different human ES cell lines surveyed by microarray. We noted that most genes detected by microarray were expressed at high tpm indicating that MPSS is more sensitive than microarray analysis. MPSS however appeared to be able to identify genes detected by microarray. Analysing an additional 400 markers detected by MPSS using focused microarray or RT-PCR confirmed their expression [3], (data not shown). Likewise, MPSS analysis showed good concordance with the EST scan data at a fraction of the price. In contrast to the EST scan, tpm levels determined by MPSS are highly correlated to the mRNA levels present in the cells, even at low tpm values [25], and (Lynx unpublished results). Due to the low sampling number of most EST scans, this is not true for relatively low number of EST's found for a particular gene, and can be used only as a rough estimate of gene expression. Unlike other in depth analyses, the absence of markers in MPSS runs is also a powerful control provided that the marker possesses a GATC site. The chromosomal distribution of the genes expressed in hESC did not reveal any bias for a particular chromosome or chromosomal region. While a couple of "hotspots" and several "cold spots" were identified, in no case was any region comprised of all transcribed or all silent genes.

Another important conclusion from our analysis is that selection of input RNA is critical. In our case we tested samples repeatedly to assess their purity and made considerable efforts to establish subclones that did not require feeder cells that could be potentially contribute transcripts to the analysis. Given the range of tpm of biologically relevant molecules (5 to 32,000 in this experiment) we predict that even a 5% contamination can confound results or detailed comparisons across different laboratories.

We note also that gene transcription from both the X and Y chromosome is observed indicating that at least subtle differences will exist between male and female lines even in the undifferentiated state. Sex-based gene expression, along with MHC gene expression and ratio of expression of imprinted genes could serve to distinguish between different ES cell populations. The present results further suggest that analysing embryoid bodies that differentiate stochastically or analysing tissue samples (with variable proportions of cells) by MPSS will prove more difficult and that results will be variable. We suggest that variability can be reduced by pooling samples, normalizing by careful testing for known markers of differentiation, by semi quantitative PCR, or by focused microarray analysis.

While MPSS is cost-effective and sensitive, it is by no means perfect. MPSS is limited by the requirement that DpnII sites (GATC) be present in a gene and be present in a unique locus such that the signature obtained is unique. For example, SNRF expression could not be assessed directly, as no GATC site is present. The signatures for ZFP42 are ambiguous and map to multiple transcripts. Although MPSS can distinguish between alternate transcript termination sites, MPSS cannot distinguish between alternative splicing events and possible incomplete digestion during the sample preparation process. Signature lengths are relatively short and it is possible to have to select between multiple genome hits (reviewed in [16]. Sequencing is performed four bases at a time and transcripts that contain palindromic sequences (in particular double palindromes) are often undetected because of self-hybridization of single DNA strands on the bead. A survey of the genome suggests that this is a rare event (approximately 3% of all virtual signatures in human MGC database have double palindromes). The NODAL gene is an example for such an event, where the class 1 signature was lost and NODAL expression is detected only by a signature resulting from incomplete digestion during library construction (see results). The success of MPSS analyses also depends to a large extent on the quality of genomic information available and, in our opinion, currently is best utilized to analyse human cells. Furthermore, MPSS itself may not be the best method for routine, lower throughput analyses, given price per sample, sample processing time and the large amount of data generated, which requires considerable analysis. However, the database, once developed, is extremely valuable provided it is freely available to make comparisons and to select subsets of genes for further analysis. MPSS information can be effectively utilized by establishing a common database of markers expressed at a defined stage in the differentiation of cells. Additional data sets from sampling of cells at well-controlled stages of differentiation that can be readily accessed and compared to existing datasets will provide the most information while still being cost effective. The genome database is an example of such sharing that has proven to be an invaluable resource for our experiments. Such a strategy requires cooperative pooling of information and free sharing such that individual results can be readily compared against validated datasets. Our future experiments will be directed and developing additional data sets of ES cell differentiation, which can be shared in a manner similar to the present set.

## Conclusions

Our results provide a comprehensive data set that can be effectively utilized to analyse expression patterns of known and unknown genes. Comparison with other data sets provides independent confirmation of results and shows a high level of concordance. The caveats to all such large-scale comparisons are discussed and the importance of pooling data and comparing across multiple data sets is demonstrated.

## Methods

### Cell culture

The human ES cell lines H1, H7, and H9 were maintained under feeder-free conditions in MEF-conditioned medium supplemented with bFGF as described previously [19,26].

### MPSS

MPSS was performed using RNA from three pooled ES cell lines (H1, H7, and H9) that had been maintained in feeder free culture conditions and evaluated for the presence of ES cell markers and absence of markers of differentiation. The mRNA was converted to cDNA and digested with DpnII. The last DpnII site and the downstream 16 bases were cloned into Megaclone vectors and their sequences determined according to the MPSS protocol [15,16,25]. A total of 2.786.765 sequences were read from four different runs and 48,388 unique signatures were identified. The abundance for each signature was converted to transcripts per million (tpm) for the purpose of comparison between samples. Signatures at an abundance of less than 4 tpm or those that were not detected in at least two runs were removed and a total of 22,136 sequences were analyzed further. All data is available for download from Lynx [27]

### MPSS signature classification and annotation

To generate a complete, annotated human signature database, we extracted all the possible signatures ("*virtual signatures*") from the human genome sequence, the human Unigene sequences, and human mitochondrion. Each virtual signature was ranked, as outlined in the table 1a, based on its position and orientation in the original sequence. Unigene, genomic, and mitochondrial hits were combined and grouped by signature. The annotation was then assigned to the signature in following order of preference: repeat warnings (signature hits more than 100 genome locations); mitochondrial hits; Unigene hits; genome hits (if no transcript match found). If a signature matched only one Unigene cluster, the MPSS signature class is the lowest class of the member sequences of the cluster. If a signature hits multiple Unigene clusters, the best cluster hit is selected based on the lowest MPSS signature class or the largest number of member sequences. The resulting signature database was used to annotate the data from the experiments Initially the signatures were annotated using genome version hg15 (April 2003, Golden Path, UCSC,) and Unigene build #161 (additional file 2). Recently we re-annotated all signatures using genome version hg16 (July 2003, Golden Path, UCSC) and Unigene build #169 (additional file 3). Both annotations are available for download in supplemental tables [27].

### Microarray

Analysis was performed as described in Bhattacharya et al., [9] using six different samples. These included two lines from Bresagen (01 and 02), the pooled sample from Geron comprising feeder free subclones of (H1, H7, H9), H1, grown in our laboratory on feeders and H9 and I6 from Dr. Itskovitz-Eldor grown following their published protocols.

### EST-enumeration

EST frequency counts of genes expressed in human ES cells were done as described ([8]). Statistical significance was determined using the Fisher Exact Test [28].

### Chromosomal mapping of MPSS signatures and UniGene clusters to the human genome

MPSS signatures with a hit to a UniGene cluster were mapped to the Giemsa staining cytobands of the hg15 release of the human genome (April 10, 2003 freeze, [29]). By this method, 7731 MPSS signatures were mapped to the cytobands of the human genome. Similar mapping was done for all UniGene clusters for which the chromosomal mapping is known. In order to achieve a gene-based rather than a transcript (i.e. splice variant) based distribution of genes splice variants the UniGene clusters were filtered using LocusLink data [30], since LocusLink captures all characterized splice variants of a particular gene. 23,828 UniGene clusters were identified by this method and mapped to the cytobands of the human genome. To discover differences in the number of genes mapped to each cytoband, the number of genes mapped to each cytoband was compared to the total number of genes analyzed, for both the MPSS signatures as well as for the UniGene clusters. The Fisher test [28] was used to determine the statistical significance, using a p-value = 0.05 as cutoff.

### Gene detection by RT-PCR

Total RNA was isolated from cell pellets using RNAeasy Qiagen mini protocol and kit. cDNA was synthesized using 100 ng of total RNA in a 20-μl reaction. Superscript II (Gibco-BRL), a modified Maloney murine leukemia virus RT, and Oligo (dT)12–18 primers were used according to the manufacturer's instructions (Gibco-BRL). The list of primers used for RT-PCR and annealing conditions are described previously [3]).

## Authors' contributions

RB, IK and MR were primarily responsible for the data analysis and writing and editing the manuscript. ST generated the ES cell samples and verified their quality, TM performed the RT-PCR and JC performed the immunocytochemistry. RP generated the microarray data and TV and JL provided support and supervision for RB, IK, ST.

## Supplementary Material

Additional File 1 The document describing details of MPSS analysis of the HuES cells performed at Lynx, and algorithm for initial MPSS signature annotation and classification. Click here for file

Additional File 2 The file contains MPSS data for 22,136 significant and reliable signatures, annotated with genome version hg15 (April 2003, Golden Path, UCSC) and the human Unigene build Hs.161. Click here for file

Additional File 3 The file contains MPSS data for 22,136 significant and reliable signatures, annotated with genome version hg16 (July 2003, Golden Path, UCSC) and the human Unigene build Hs.169. Click here for file

Additional File 4 table containing data for 8679 unigene clusters, 11 mitochondrial genes, and including 1991 signatures that did not map to unigene but uniquely matched genomic sequences (potential novel transcripts). Click here for file

Additional File 5 Top 200 rows from the table HuES17_onetpmHs169hg16.xls. Click here for file

Additional File 16 List of tpm of genes identified by Sperger et al [11] present in the MPSS dataset. Click here for file

Additional File 17 List of tpm of genes identified by Richards et al [8] present in the MPSS dataset. Click here for file

Additional File 18 List of tpm of genes identified by MPSS in the BG02 dataset. Click here for file

Additional File 6 This file lists the genes located on the X and Y chromosome for which a MPSS signature sequence was found in the HuES cells. The X and Y chromosome genes are listed in separate worksheets. Chromosome: X or Y chromosome Cytoband: Giemsa staining cytoband Signature: MPSS signature sequence Tpm: mean abundance for a signature derived from all MPSS runs for the sample, in transcripts per million Seq_id: Repeat if hit genome more than 100 locations; UniGene cluster ID if hit one or more UniGene clusters; Chromosome number if hit one genome location; MultiGenome if hit genome multiple times; Description: description of annotation (see Appendix B for more details). Click here for file

Additional File 7 List of all UniGene clusters (release 161) located on the X and Y chromosome. The X and Y chromosome genes are listed in separate worksheets. Chromosome: X or Y chromosome Cytoband: Giemsa staining cytoband Locusid: Locus Link identifier UniGene cluster: UniGene cluster ID Description: description derived from UniGene Click here for file

Additional File 8 Graphical representation of hotspots and coldspots for all human chromosomes. Cytobands with significantly higher number of genes in HuES cells are marked with '+' and cytobands with lower number of genes in HuES cells are marked with '-'. The number of '+' or '-' signs is proportional to the difference, with one '+' or '-' representing the fold difference. For example '++' represents a 2-fold higher number of genes expressed in HuES cells compared to the expected number based on the number of genes known to be located in the same cytoband. Click here for file

Additional File 9 List of all chromosal regions that appeared statistically distinct. Click here for file

Additional File 10 List of genes expressed in HuES cells and located in the hotspots and coldspots. Chromosome: X or Y chromosome Cytoband: Giemsa staining cytoband Signature: MPSS signature sequence Tpm: mean abundance for a signature derived from all MPSS runs for the sample, in transcripts per million Seq_id: Repeat if hit genome more than 100 locations; UniGene cluster ID if hit one or more UniGene clusters; Chromosome number if hit one genome location; MultiGenome if hit genome multiple times; Description: description of annotation. Click here for file

Additional File 11 List of all UniGene clusters located in the hotspot and coldspot regions. Chromosome: X or Y chromosome Cytoband: Giemsa staining cytoband UniGene ID: UniGene cluster ID Locusid: Locus Link identifier Description: description derived from UniGene; Click here for file

Additional File 12 This file contains the mapping information of MPSS signatures to the EST scan genes for major signaling pathways. Each pathway is presented in a separate worksheet Columns: Locusid: Locus Link identifier Pathway: Pathway the gene has been assigned to. Signature: MPSS signature sequence Tpm: mean abundance for a signature derived from all MPSS runs for the sample, in transcripts per million Stdev: standard deviation of the mean abundance from multiple MPSS runs Hit genome: HitGenome – Numbers of genomic locations a signature maps to HitUniGene: Numbers of UniGene Clusters a signature maps to; Seq_id: UniGene cluster ID ('Hs.' Has been omitted). NULL if there is no MPPS signature but an EST from the EST scan. Class: signature class (see Appendix B for more details); Title: description, derived from UniGene Click here for file

Additional File 13 This file contains additional lists of potential homeodomain proteins, genes of unknown function, zinc finger proteins in separate worksheets. Tpm: mean abundance for a signature derived from all MPSS runs for the sample, in transcripts per million Stdev: standard deviation of the mean abundance from multiple MPSS runs Hit genome: HitGenome – Numbers of genomic locations a signature maps to HitUniGene: Numbers of UniGene Clusters a signature maps to; Seq_id: UniGene cluster ID. Class: signature class (see Appendix B for more details); Title: description, derived from UniGene. Click here for file

Additional File 14 List of tpm of potential 400 ES enriched genes in other cell lines examined. Click here for file

Additional File 15 List of all tpm of potential 13 ES novel genes in all tissues and cell lines examined. Click here for file
