# Supplementary material for: MPSS profiling of human embryonic stem cells
Source: BMC Dev Biol. 2004 Aug 10;4:10. doi: 10.1186/1471-213X-4-10 (PMC514702; doi:10.1186/1471-213X-4-10)
Supplement: Additional File 1 — The document describing details of MPSS analysis of the HuES cells performed at Lynx, and algorithm for initial MPSS signature annotation and classification. [file 1471-213X-4-10-S1.doc]

# MPSS Analysis of

# Human Embryonic Stem Cell RNA

**For Dr. Mahendra Rao**

National Institute of Aging

# July 1^st^, 2003

# Irina Khrebtukova, Ph.D.

# Staff Bioinformatics Scientist

# LYNX Therapeutics Inc.

# 25861 Industrial Blvd. Hayward, CA 94545

# Tel: 510 723 9219

# 1. Generation of MPSS datasets

Total RNA of human embryonic stem cells was received from National Institute of Aging (NIA) and analyzed through the Bioanalyzer, passed our quality control test and qualified for the analysis (see Appendix C. Sample QC Report). The mRNA was processed according to the MPSS protocol as outlined in the accompanying publications (Brenner *et al.* 2000a; Brenner *et al.* 2000b). Briefly, the mRNA was reverse transcribed and the cDNA was digested with Dpn II. The 21 bases adjacent to the 3’ most Dpn II site was cloned into a Megaclone vector. The resulting library was amplified and loaded onto microbeads. About 1.6 million microbeads were loaded into each flow cell (See MPSS tables below for each flow cell result) and the signature sequences were determined by a series of enzymatic reactions as outlined in the above publications. The abundance for each signature was converted to transcripts per million (tpm) for the purpose of comparison between samples. The information about library construction (Cell/tissue table and the cDNA library table), the MPSS runs (MPSS table), and the MPSS results (Run group table) are shown in appendix A.

**2. Signature Annotation**

# To generate a complete, annotated mouse signature database, we extracted all the possible signatures (“*virtual signatures*”) from the human genome sequence (Release hg15, April 2003) and the human UniGene sequences (UniGene build #160). Each virtual signature is ranked, as outlined in Appendix B, based on its position and orientation in the original sequence. The annotation for that sequence is then assigned to the signature and the resulting signature database is used to annotate the data from the experiments, using our “TopHit” algorithm (see more in Appendix B).

**3. Results**

The results are presented in both Excel table (HuEs_tophit.xls) and in tab-delimited text format (HuEs_tophit.txt).

**Table column description**

Signature – self-explanatory;

TPM – mean abundance for a signature derived from all MPSS runs for the sample, in transcripts per million;

STDEV – standard deviation of the mean abundance from multiple MPSS runs;

HitGenome – Numbers of genomic locations a signature maps to;

HitUniGene – Numbers of UniGene Clusters a signature maps to;

Sequence_id – Repeat if hit genome more than 100 locations;

UniGene cluster ID if hit one or more UniGene clusters;

Chromosome number if hit one genome location;

MultiGenome if hit genome multiple times;

Links – http links to Unigene and USCS genome browser;

Class – signature class (see Appendix B for more details);

Description – description of annotation (see Appendix B for more details);

**Appendix A. Sample, Library Construction and MPSS Run Information**

# HuEs.norm

#

| Cells/tissue | |
| --- | --- |
| Library | HuES.norm_sig01 |
| Cell type | Human embryonic stem cells |
| Source | National Institute of Aging (NIA) |
| RNA isolation | NIA: Isolation of total RNA  LYNX: Isolation of mRNA |

| cDNA library | |
| --- | --- |
| Library | DpnII restriction - (signature cloning using MmeI) |
| Sequence length | 17bp |

| MPSS | | | | |
| --- | --- | --- | --- | --- |
| Runs | Date | Beads | Signatures | QC |
| HuES.norm_sig01.4625F.a | Jun 15, 2003 | 693,000 | 26,537 | Passed |
| HuES.norm_sig01.4625F.b | Jun 15, 2003 | 781,726 | 26,610 | Passed |
| HuES.norm_sig01.4625T.a | Jun 19, 2003 | 687,225 | 26,583 | Passed |
| HuES.norm_sig01.4625T.c | Jun 25, 2003 | 624,814 | 23,383 | Passed |

| Run group | | |
| --- | --- | --- |
| Total Beads  successfully sequenced | Total distinct Signatures | Significant and Replicate Signatures  (delivered) |
| | 2,786,765 | | --- | | 48388 | 22136 |

Significant – signatures detected at abundance >3 tpm;

Replicate – signatures found at least in two replicate MPSS runs.

# Appendix B. Signature Classification and Annotation

(1) Criteria used to classify cDNA signatures

A. The position of the signatures relative to polyadenylation signals and poly-A tails.

- Polyadenylation signals are any sequences matching to AWTAAA (AATAAA or ATTAAA) that occur within the last 155 bases of the cDNA sequence.
- Poly-A tails are 15 base sequences containing >= 12 As that occur within the last 114 bases of the sequence.

B. The orientation of the signatures relative to the 5' to 3' direction of the source mRNA.

- The source sequence is scanned first in its forward direction and then in it's reverse complement.
- If the sequence's accession starts with NM designation, or its description contains 'complete cds', the forward read of the sequence is considered as the 5' to 3' direction.
- If the sequence has a Poly-A signal and tail in its forward or reverse read, this read is considered as the 5' to 3' direction.
- In all other circumstances, the 5' to 3' direction is considered as “unknown”.

(2) Classification of cDNA signatures

| **Virtual Signature Class** | **mRNA Orientation** | **Poly-Adenelation Features** | **Position** |
| --- | --- | --- | --- |
| 0 | Either - Repeat Warning | Not applicable | Not applicable |
| 1 | Forward Strand | Poly-A Signal, Poly-A Tail | 3' most |
| 2 |  | Poly-A Signal | 3' most |
| 3 |  | Poly-A Tail | 3' most |
| 4 |  | None | 3' most |
| 5 |  | None | Not 3' most |
| 11 | Reverse Strand | Poly-A Signal, Poly-A Tail | 5' most |
| 12 |  | Poly-A Signal | 5' most |
| 13 |  | Poly-A Tail | 5' most |
| 14 |  | None | 5' most |
| 15 |  | None | Not 5' most |
| 22 | Unknown | Poly-A Signal | Last before signal |
| 23 |  | Poly-A Tail | Last before tail |
| 24 |  | None | Last in sequence |
| 25 |  | None | Not last |
| 1000 | Unknown - Derived from Genomic Sequence | Not applicable | Not applicable |

(3) Selection of the top hit and annotation

UniGene hits, Genomic hits, and Mitochondrial hits are combined and grouped by signature. Each signature’s hits are sorted by the following criteria and then the first in the list is chosen:

1: Repeat warnings (signature hits >100 genome locations)

2: Mitochondrial hits

3: UniGene hits

4: Genomic hits (signature hits <=100 genome locations)

**Repeat warnings**

If a signature hits more than 100 genome locations, these hits are merged into a single repeat warning ‘hit’ with class = 0, sequence id = ‘REPEAT’ and description “*** WARNING >100 Genome Hits ***”.

### Mitochondrial hits

If a signature hits mitochondrion, the sequence ID is the gene name; the description will indicate the functional annotation for the region hit.

### UniGene hits

If a signature hits only one UniGene cluster, the class is the lowest class of the member sequences of the cluster; the sequence ID is the UniGene cluster ID; the description is the concatenation of

1: Accession for the lowest class hit in cluster.

2: Cluster id.

3: Description of the cluster.

If a signature hits multiple UniGene clusters, the best cluster hit is selected based on the criteria in the following orders:

1: The lowest class.

2: The largest number of member sequences.

### Genome hits

If a signature hits only one genome location, the class is 1000; the sequence ID is the chromosome number; and the description gives the exact genome location.

If a signature hits 2 to 100 genome locations, they are combined into a single hit with class = 1000, sequence id = ‘multiGenome’ and description “xxx genome locations”.
